# Biocontrol Ability Against Harmful Microbial Contamination of Vegan Mortadella with an Ingredient of Oat Fermented by *Lactiplantibacillus plantarum*

**DOI:** 10.3390/foods14132195

**Published:** 2025-06-23

**Authors:** Ana Moreno, Alberto Gonçalves, Mario Riolo, Victor Dopazo, Jorge Calpe, Giuseppe Meca

**Affiliations:** 1 Laboratory of Food Chemistry and Toxicology, Faculty of Pharmacy, University of Valencia, Av. Vicent Andrés Estellés s/n, 46100 Burjassot, Spain; ana.moreno@uv.es (A.M.); jorge.calpe@uv.es (J.C.); giuseppe.meca@uv.es (G.M.); 2 Graduate Program in Animal Science, Pontifícia Universidade Católica do Paraná, Rua Imaculada Conceição, 1155 Prado Velho, Curitiba 80215-901, PR, Brazil; alberto.evangelista@pucpr.edu.br; 3 Department of Agriculture, Food and Environment (Di3A), University of Catania, Via Santa Sofia 100, 95123 Catania, Italy; mario.riolo@unict.it

**Keywords:** biopreservation, vegan food, lactic acid bacteria, fungal contamination, foodborne pathogens

## Abstract

The rising demand for vegan products calls for new plant-based antimicrobial preservation methods. This study evaluates an antifungal ingredient obtained by fermenting oat drink with lactic acid bacteria to extend vegan mortadella’s shelf life. In vitro tests showed antimicrobial effects against *Aspergillus flavus*, *Penicillium commune*, and *Listeria monocytogenes* (inhibition zones: 2–5 mm). The enrichment of the oat drink culture medium with additional nutrients enhanced fermentation performance and increased antifungal activity. The fermented culture medium with the highest antimicrobial activity was used to develop a bioactive ingredient for the preservation of vegan mortadella conservation. Adding 3% of this ingredient to vegan mortadella improved microbial stability, reducing mesophilic bacteria by 2.5 Log_10_ CFU/g and increasing lactic acid bacteria. Lower pH and water activity changes were observed but remained within quality standards. Contamination assays showed a consistent reduction of *A. flavus* over 7 days, while *P. commune* and *L. monocytogenes* dropped below detection within 2 days. In contrast, control samples maintained contamination levels near 3.0 Log_10_ CFU/g. These findings support the potential of fermented oat-based ingredients as effective, natural preservatives for vegan foods.

## 1. Introduction

The growth of vegan meat alternatives reflects a significant transformation in dietary patterns, driven by ethical, environmental, and health motivations. These plant-based products aim to replicate the sensory and nutritional characteristics of conventional meats while avoiding animal-derived ingredients [1]. Notably, efforts to mimic traditional sausages have extended to mortadella, resulting in novel vegan mortadella-type products designed to closely resemble the sensory and structural characteristics of the Italian original [2]. Among the various components used in their formulation, oats have gained prominence due to their nutritional profile, sustainability, and functional properties [3]. Oats provide abundant dietary fiber, protein, and bioactive compounds like β-glucans, making them a powerful addition to vegan formulations including beverages, fermented products, and meat analogues [4,5].

However, like all minimally processed or ready-to-eat foods, vegan meats are susceptible to microbial contamination throughout their production chain [6]. This includes pathogenic bacteria, such as *Listeria monocytogenes*, and spoilage molds like *Aspergillus* and *Penicillium* species [7]. *L. monocytogenes* is particularly problematic due to its ability to grow at refrigeration temperatures and cause severe illness in vulnerable populations [8]. Additionally, filamentous fungi degrade sensory quality and present a toxicological hazard through mycotoxin production [9,10]. These concerns are compounded by the clean-label expectations of consumers, which limit the use of synthetic preservatives and emphasize the need for natural preservation strategies [11]. In addition, oat fermentation by lactic acid bacteria has been shown to enhance the prebiotic qualities of oats, broadening their spectrum of bioactivities. This further establishes oats as an excellent medium for LAB fermentation, enhancing their antimicrobial potential [12].

Biopreservation using lactic acid bacteria (LAB) has emerged as a promising alternative to synthetic additives [13]. LAB produces antimicrobial effects through multiple mechanisms, including acidification, competition for nutrients, and the production of bioactive metabolites such as bacteriocins, reuterin, and phenyllactic acid [14]. In foods, LAB can be incorporated during production or applied as protective cultures in packaging [15,16]. These compounds are effective against a broad spectrum of foodborne pathogens and spoilage organisms, including *L. monocytogenes* and molds [17,18]. Additionally, the antimicrobial properties persist despite industrial processes such as increased temperature and enzyme presence [19].

Moreover, the application of LAB fermentation aligns with sustainable practices. It can utilize plant-based byproducts such as citrus peels to create bioactive formulations [20]. This dual function, not only enhancing food quality but also minimizing waste, directly addresses the rising demand for sustainable food alternatives [21]. Based on this evidence, the objectives of this study were to: (a) develop and assess the antimicrobial potential of a bioactive ingredient produced through lactic acid bacterial fermentation of vegetable sources; (b) investigate how different concentrations of the antifungal ingredient affect the technological properties of a vegan mortadella; and (c) evaluate the antimicrobial properties of this ingredient in vegan mortadella.

## 2. Materials and Methods

### 2.1. Chemicals and Microorganisms

Milli-Q water with a resistivity of <18 MΩ·cm was obtained using a Milli-Q purification system (Millipore, Bedford, MA, USA). Buffered peptone water, potato dextrose agar (PDA), Man, Rogosa, Sharpe Agar (MRSa), Man Rogosa Sharpe Broth (MRSb), brain heart infusion (BHI) and plate count agar (PCA) were purchased from Liofilchem (Teramo, Italy). Agar, glucose (>99.9), yeast extract, soy protein, ammonium sulfate (>99.9), sodium chloride (>99.9), magnesium sulfate (>99.9), potassium phosphate (>99.9), and manganese sulfate (>99.9) were purchased from Sigma-Aldrich (Dublin, Ireland). Beans and food colorant were obtained from Consum Cooperativa (Valencia, Spain).

The LAB strain isolated from vegan products used in this study was previously analysed by our group and demonstrated its potential to ferment oat drink, producing bioactive antimicrobial agents in vitro screening tests performed on several bacterial isolates. This strain was identified at the species level as *Lactiplantibacillus plantarum* (*L. plantarum*) through microbial identification and characterization by MALDI-TOF-MS (matrix assisted laser desorption/ionization time-of-flight mass spectrometry) in the Spanish Type Culture Collection (CECT), Valencia, Spain [22].

Microbial isolates including *Aspergillus fumigatus* CECT 20827, *Aspergillus niger* CECT 2088, *Aspergillus steynii* CECT 20510, *Penicillium commune* CECT 20767, *Penicillium digitatum* CECT 2954, *Penicillium expansum* CECT 2278, *Penicillium camemberti* CECT 2267, *Penicillium italicum* CECT 20332, *Penicillium nordicum* CECT 2320, *Penicillium roqueforti* CECT 2905, and *Listeria monocytogenes* CECT 935 were obtained from the CECT collection. *Penicillium verrucosum* D-01847 VTT was acquired from the VTT Technical Research Centre of Finland (Espoo, Finland), and *Aspergillus flavus* ITEM 8111 from the ISPA Culture Collection (Bari, Italy).

Oat drink was produced according to the method described by Demïr et al. (2021) with minor modifications [23]. Oat was combined with distilled water (1:5 *w*/*v*), heated to 95 °C, and soaked for 15 min to enhance extraction. The mix was homogenized with a blender (John Oster Manufacturing Company, Racine, WI, USA) and filtered through a cheesecloth to obtain the oat drink.

### 2.2. Preparation, Fermentation and Characterization of the Oat Drink Culture Mediums

A total of 3 different culture mediums for the LAB fermentation were prepared following the steps described in Dopazo et al. (2023) with adaptations for this study [24]. The mediums produced for this study were: (1) Culture medium oat drink (OD). (2) Culture medium oat drink enriched with glucose (ODG): oat drink complemented with 10.0 g/L of glucose. (3) Culture medium oat drink enriched with nutrients (ODN): oat drink with 10.0 g/L of glucose, 10.0 g/L of yeast extract, 10.0 g/L of soy protein, 6.5 g/L of sodium chloride, 2.5 g/L of dipotassium phosphate, 0.25 g/L of magnesium sulphate, and 62.5 mg/L of manganese sulphate. After homogenization and pasteurization for 30 min at 80 °C, the culture mediums were cooled to 30 °C. Finally, they were inoculated at a concentration of 5% (*v*/*v*) with a LAB suspension, from a previous incubation of 24 h at 37 °C in MRSb and then washed twice with a PBS solution 0.1 M. Afterwards, the culture mediums were incubated at 37 °C for 48 h.

For a better evaluation of the fermentation of the culture a comprehensive assessment of key parameters for effective LAB fermentation and antifungal activity was conducted. The pH of the samples was analysed using a XS pH 7 Vio pH-meter with a 2 Pore Steel T electrochemical sensor (XS Instruments, Carpi, Italy). Bacterial count (performed in MRS), lactic acid and phenyllactic acid measurement was performed according to the steps described in Dopazo et al. (2023) [24].

The resultant fermented culture medium was frozen at −20 °C. Subsequently, the samples underwent lyophilization in a FreeZone 2.5 L freeze-drying equipment (Labconco, Kansas City, MO, USA) for further analysis [25].

### 2.3. Evaluation of the Antimicrobial Properties of the LAB Fermented Oat Drink Culture Mediums

The qualitative antifungal properties of the oat drink culture mediums after the fermentation were evaluated by the agar diffusion method [26]. First, a suspension of 400 g/L of the samples was prepared using sterilized distilled water. For the antifungal assays, PDA plates were used for the assays against fungi, while BHI agar plates were used for *Listeria monocytogenes*. Microbial suspensions were spread on the agar plates with a sterile swab, and wells were pierced in the agar using sterile 1 mL micropipette tips. Subsequently, 100 µL of the sample suspension was added to each well. After a 48-h incubation at 30 °C, inhibition zones were measured to evaluate antifungal activity. Each sample was compared to a control containing a non-fermented culture medium to assess relative antifungal effects. Assays were performed per triplicate and the mean was used as a result.

### 2.4. Production of the Vegan Mortadella Incorporating the Bioactive Ingredient

The culture medium ODN fermented by the LAB isolated evidenced the best results in the antimicrobial assays and was selected for the production of the vegan mortadella bioactive ingredient. To prepare the mortadella, beans were boiled for 40 min until soft, then blended with salt, thyme and garlic into a mash. Simultaneously, an agar suspension in distilled water was heated to boiling and gently stirred for 5 min. The agar was quickly combined with the bean mash, a total of 0.150 mL of pink food colorant, and the mixture was poured into a mold and refrigerated at 5 °C for 8 h.

The final composition of the mortadella included the following ingredients: bean mash, water, agar, salt, thyme and garlic. Four variations of mortadella were prepared: a control and 3 sample versions containing the lyophilized powder from the bioactive ingredient at 0.5% (M 0.5%), 1.0% (M 1%), and 3.0% (M 3%) concentration (*w*/*w*). The bioactive ingredient was incorporated by replacing an equivalent portion of the bean mash. All mortadellas reached a final weight of 750 g. The ingredient concentrations are detailed in Table 1 for reference.

### 2.5. Evaluation of the Effect of the Bioactive Ingredient in the Vegan Mortadella Quality

To investigate the influence of the fermented ODN ingredient in vegan mortadella, several analyses were conducted through the 7-day study. The pH of the samples was measured using a XS pH 7 Vio pH-meter with a 2 Pore Steel T electrochemical sensor (XS Instruments, Carpi, Italy) [25]. Water activity (Aw) was assessed using a Humimeter RH2 water activity meter (Max-Schaller-Straße, Styria, Austria) [27]. Moisture content was determined by an indirect gravimetric method, where approximately 10 g of each sample was weighed before and after drying in an oven at 105 °C for 24 h [28]. To analyze microbial populations due to natural contamination, 10 g of vegan mortadella were homogenized in 0.1% (*w*/*v*) peptone water using a Masticator Classic Stomacher (IUL S.A., Barcelona, Spain). The homogenized samples were then serially diluted and plated on PCA, incubated at 30 °C for 48 h, for the total aerobic mesophilic bacteria count, and on MRSa under anaerobic conditions at 37 °C for 48 h, for the LAB count [29]. The color of the vegan mortadella was measured at different locations using a ColorQuest^®^ XE (HunterLab, Birstall, Leicestershire, UK) to determine hue, saturation, and intensity (HSI), comparing any differences [30]. All assays were performed in triplicate.

### 2.6. Preparation of Microbial Inoculum for In Situ Antimicrobial Activity Assays

The *Aspergillus* and *Penicillium* isolates were cultured on PDA at 25 ± 1 °C in the dark until the mycelium completely covered the surface of the Petri dish. For each isolate, a conidial suspension (10^7^ spore/mL) was prepared in sterile distilled water. The conidial concentration was determined using a Neubauer counting chamber (BLAUBRAND^®^, Merck KGaA, Darmstadt, Germany). The bacterial suspension of *Listeria monocytogenes* was prepared by cultivating the strain on BHI medium, at 37 °C for 24 h. After centrifugation at 5000× *g* for 10 min at 4 °C, bacterial cells were resuspended in sterile distilled water to achieve a final concentration of 10^8^ CFU/mL, verified using a Neubauer counting chamber. Finally, solutions were adjusted to a concentration of 2 × 10^6^ CFU/mL for the assays [31,32].

### 2.7. Assessment of the Antimicrobial Properties of the Bioactive Ingredient in the Vegan Mortadellas

The in situ antimicrobial activity of the bioactive ingredient was evaluated by monitoring fungal and bacterial contamination in mortadella samples, using both spoilage and pathogenic microorganisms throughout the storage period. The experimental procedure followed the methodology described by Dopazo et al. (2022), with adaptations specific to this study [33]. Mortadella slices of approximately 20 g were cut, and 10 µL of the microbial suspension (2.5 × 10^6^ CFU/mL) of the contaminating microorganisms was applied to four separate spots on each slice. The slices were then incubated under sterile conditions at 10 °C.

Microbial population dynamics were monitored after 0, 2, 4, and 7 days of incubation. Quantification was performed by homogenizing the samples in 0.1% peptone water using a stomacher, followed by serial dilutions and plating on agar. For *Aspergillus niger* and *Penicillium commune*, the samples were plated on PDA and incubated at 25 °C for 48 h. For *Listeria monocytogenes*, the dilutions were plated on BHI agar and incubated at 37 °C for 48 h. All assays were conducted in quadruplicate to ensure reproducibility.

### 2.8. Statistical Analysis

Statistical analyses were performed using a one-way ANOVA to evaluate differences among the experimental groups. When significant differences were detected (*p* < 0.05), Tukey’s HSD post hoc test was applied to identify specific pairwise differences. For the culture assays, six groups were compared: the three non-fermented culture media and the three fermented culture media. The culture media tested were OD, ODG, and ODN. For the vegan mortadella samples, four groups were analyzed: the control (mortadella without the bioactive ingredient) and three treatments containing 0.5%, 1.0%, and 3.0% of the bioactive ingredient (M 0.5%, M 1.0%, and M 3.0%). All statistical analyses were performed using InfoStat 2019 software (Universidad Nacional de Córdoba, Córdoba, Argentina).

## 3. Results and Discussion

### 3.1. Assessment of Fermentation Parameters and Antimicrobial Properties of Bioactive Ingredients

The data obtained from the analysis of the fermented oat mediums can be appreciated in the Table 2. The pH evaluation clearly indicated active bacterial fermentation in the LAB fermented samples. In the non-fermented control, the pH remained significantly higher, with values close to pH 7. In contrast, fermentation reduced the pH to an average of 3.46 in the inoculated samples. These results were consistent with the data obtained from other analyses. No bacterial growth was detected in the control samples based on CFS counting, and both lactic acid and phenyllactic acid were below the limit of detection. In the fermented samples, LAB counts performed in MRSa reached 7.37, 8.20, and 8.32 log_10_ CFU/mL in OD, ODG, and ODN, respectively. Lactic acid concentrations were 14.84, 17.27, and 17.34 g/L in fermented OD, ODG, and ODN, respectively. When comparing these values, both bacterial counts and lactic acid production were significantly higher in ODG and ODN than in OD, suggesting an improved fermentation performance in matrices with enhanced nutrient composition. Phenyllactic acid concentrations also increased significantly with the nutrient complexity of the matrix. Fermented OD reached 2.61 mg/L, ODG 5.42 mg/L, and ODN 9.23 mg/L, highlighting the positive correlation between nutrient availability and production of this antimicrobial metabolite.

The results of the qualitative evaluation of antimicrobial activity from fermented oat drink culture mediums are presented in Table 3. The data show that fermentation of the oat drink culture mediums by *Lactiplantibacillus plantarum* VC7 exhibited varying degrees of antimicrobial activity against both fungi and bacteria. ODN demonstrated the highest antimicrobial activity, followed by ODG, indicating that increased nutrient availability promotes antifungal metabolite production. Fermentation of the OD showed inhibition zones of less than 2 mm against certain *Penicillium* species (*P. commune*, *P. digitatum*, *P. expansum*, and *P. verrucosum*) and *Listeria monocytogenes*. This indicates that OD serves as a valuable nutrient source for microbial growth and antimicrobial metabolite production, but its antimicrobial efficacy is limited without nutrient enrichment. The ODN exhibited a significantly increased antimicrobial activity, in comparison to the other treatments, particularly against *Penicillium* species and *L. monocytogenes*, with inhibition zones exceeding 5 mm for *Aspergillus fumigatus*, *P. expansum*, *P. italicum*, and *P. nordicum*. None of the non-fermented control samples exhibited any antimicrobial activity against the tested microorganisms.

Vegan antimicrobial bioactive ingredients studied are limited in the literature. Among these, essential oils have been the most extensively studied [34]. For instance, clove oil has been shown to significantly reduce populations of *E. coli* strains evidencing minimal inhibitory concentrations of 2.5 mL/L [35]. Additionally, *Ocimum basilicum* (basil) essential oil has demonstrated inhibition zones up to 20 mm at concentrations near 1 g/L against fungal species like *Aspergillus niger* and *Fusarium solani*, as well as bacteria such as *Escherichia coli* and *Bacillus subtilis* [36]. However, not all essential oils exhibit effective activity against foodborne pathogens. Moreover, the use of this essential oils carries the problematic of undesired odors to the food applied, the extraction is related to high cost in organic solvents for the extraction and economically in expensive techniques [37]. The method described in this study not only yields comparable results but also offers a simpler production process. The application of fermented oat drink as a bioactive ingredient offers a promising, novel alternative to conventional additives used in the food industry for protecting against microbial contaminants.

Unlike essential oils, the antimicrobial activity of ODN is attributed to the bioactive compounds generated during fermentation by *Lactiplantibacillus plantarum*. These include organic acids, such as lactic acid, acetic acid, or phenyllactic acid, and other fermentation-derived metabolites that lower pH and disrupt microbial membranes [38]. In particular, phenyllactic acid has been associated with antifungal and antibacterial properties in cereal-based matrices [39]. Moreover, the fermentation process may enhance the release or transformation of oat phenolics and fibers, contributing further to antimicrobial activity through synergistic effects [40]. This suggests that the combination of oat and lactic acid bacteria fermentation contributes directly to the antimicrobial activity observed.

Previous studies have shown that nutrient enrichment alone does not explain the increased antimicrobial activity observed in ODN, highlighting the importance of the oat-based matrix. While nutrient-rich media such as MRS broth can support LAB growth, they often lack the structural or biochemical components required for substantial antimicrobial metabolite production [24]. Similarly, certain food matrices may not function as effective substrates for antimicrobial synthesis during short fermentation periods, likely due to limited nutrient availability that slows microbial growth. However, the addition of targeted nutrients can accelerate LAB growth and enzyme activity, enhancing the breakdown and utilization of the food matrix for metabolite production [41]. This likely explains the higher antimicrobial activity observed in the present results. Nevertheless, exploring the potential of using food matrices alone remains valuable, as reducing the need for nutrient supplementation could lower production costs and improve the scalability of antimicrobial ingredient development for industrial applications [42].

This phenomenon was also evident in the assessment of fermentation parameters, where the use of more complex matrices significantly enhanced key indicators relevant to the objectives of this study, such as bacterial growth and the presence of antimicrobial metabolites. The lack of significant differences in lactic acid production between fermented ODG and ODN can be attributed to the similar glucose content in both matrices, as lactic acid production by LAB is closely linked to glucose metabolism [43]. In contrast, the increasing complexity of the culture medium led to a higher production of secondary antifungal metabolites, particularly phenyllactic acid. The literature highlights the importance of a broad range of minor antimicrobial compounds as key contributors to the overall antifungal activity of LAB. The synthesis of these compounds is associated with the utilization of diverse nutrients, not solely disaccharides [14]. Therefore, based on the fermentation performance and antimicrobial profile, the LAB-fermented ODN demonstrated the most promising results and appears to be the most suitable candidate for use as a bioactive ingredient.

### 3.2. Assessment of the Effect of the Bioactive Ingredient in the Vegan Mortadella Quality

The results of how the bioactive ingredient affected the mortadella quality are shown in Table 4. The control consisted of mortadella without the bioactive ingredient, while M 0.5%, M 1.0%, and M 3.0% corresponded to mortadella samples formulated with 0.5%, 1.0%, and 3.0% of the bioactive ingredient derived from the ODN medium fermented by *L. plantarum* VC7, respectively. The pH displayed a consistent trend throughout the study. Following mortadella preparation, all treatments initially exhibited a rise in pH for two days, followed by a sharp decline after day 2, returning to levelssimilar to day 0. As expected, among the treatment samples, pH values declinedas the concentration of the bioactive ingredient in the mortadella increased. It can be observedthat the values from the control sample fluctuated within 5.93 to 6.22, while in the sample with a 3% of the bioactive ingredient values were between 4.60 and 4.85 (Table 4). The water activity of the samples was affected differently. In general, the greatest values were found at day 0 and 3, while lower values were recorded on days 2 and 7. The comparison of the samples evidenced a decrease in the Aw value as the concentration of the ingredient increased. The samples reached average Aw of 0.95, 0.91 and 0.90 in the M 0.5%, M 1.0%, M 3.0%, respectively, while in the control the value was 0.95 on average (Table 4). The moisture content was significantly greater in the samples with a higher concentration of the ingredient, starting at moistures of 30 g/100 g in sample M 3.0%, a total of 5 g/100 g greater than in the control. Nevertheless, after day 3 the moisture concentration in the samples began to significantly decline in the treatment samples losing 2.7, 2.3 and 4.1 g/100 g when comparing the initial measures with the ones after a 7 day observation. Meanwhile, the control samples increased the moisture content 3.3 g/100 g over the same period of time (Table 4). Finally, the HSI values were lower in the control in comparison to the samples, the difference among samples were minimal. Average values for sample treatments were above 48 while in the control the average HSI was 26. During incubation, HSI values decreased consistently from day 0 to day 7 for all samples. The highest decrease was observed in the samples M 1.0%, of 11 units of HSI, and the lowest in the sample M 3.0%, of 4 units of HSI (Table 4).

**Figure 1:**
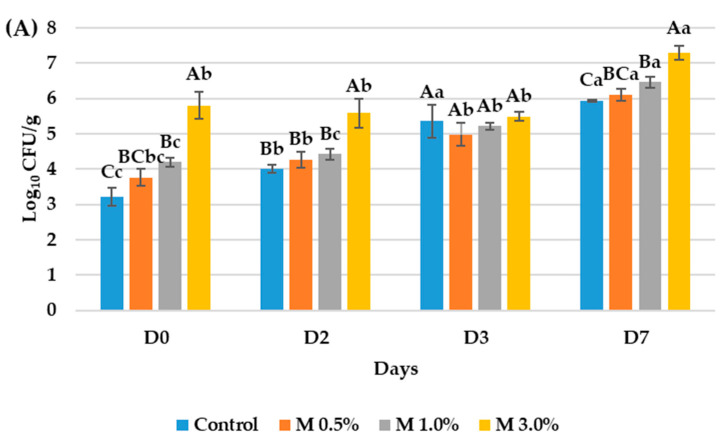

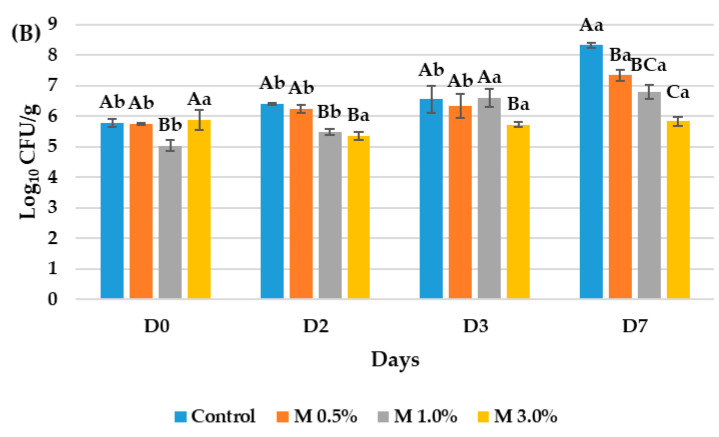
Results from the quantification of the Log_10_ CFU/g of food in (**A**) lactic acid bacteria count and (**B**) total aerobic mesophilic bacteria count of the mortadella samples thought the 7-day incubation. The control consisted of mortadella without the bioactive ingredient, while M 0.5%, M 1.0%, and M 3.0% corresponded to mortadella samples formulated with 0.5%, 1.0%, and 3.0% of the bioactive ingredient derived from the ODN medium fermented by *L. plantarum* VC7, respectively. Different letters were used to signal significative differences of the studied parameters. Capital letters were used for the comparation among different days and case letters for the comparation among samples the same day.

Quantification of Log_10_ colony forming units per gram of food (CFU/g) can be appreciated in Figure 1. It indicated that lactic acid bacterial populations growing under anaerobic conditions at 37 °C increased significantly in both the control and treated samples during the 7-day incubation. The final concentration in the control samples was initially of 3.31 Log_10_ CFU/g and ended at 5.93 Log_10_ CFU/g. The initial concentration of the samples M 0.5%, M 1.0%, and M 3.0% were 3.76, 4.19, and 5.80 Log_10_ CFU/g, initially, and ended at 6.10, 6.46, and 7.29 Log_10_ CFU/g, respectively. The average microbial growths during the days of the analysis indicated that the highest rise in bacterial concentration was observed in the control, 0.91 Log_10_ CFU/g, while the lowest was detected at the sample M 3.0%, of 0.50 Log_10_ CFU/g (Figure 1A). Throughout the MRSa study, samples with a higher concentration of the bioactive ingredient exhibited higher bacterial populations, likely due to the presence of lactic acid bacteria associated with the ingredient. In contrast, mesophilic aerobic bacterial counts on PCA showed a decrease in bacterial populations when the concentration of the antimicrobial ingredient increased. Specifically, samples containing 3.0% of the ingredient maintained a close to stable cell counts over the 7-day period, between 5.36 to 5.87 Log_10_ CFU/g, while lower ingredient concentrations were associated with an increase in Log_10_ CFU over time. The biggest increase was detected in the control samples rising from the 5.78 to 8.32 Log_10_ CFU/g (Figure 1B).

**Figure 2:**
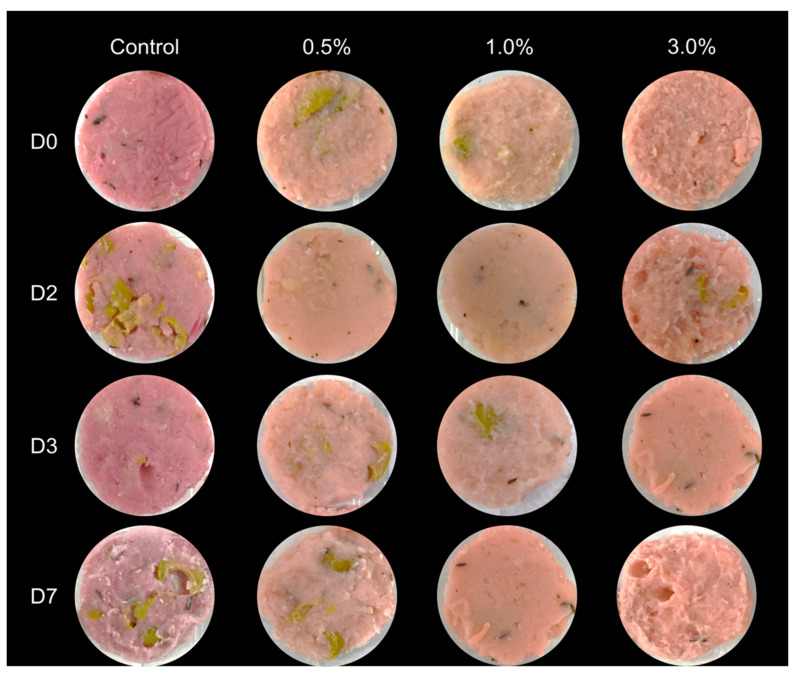
Mortadella control and mortadella samples with 0.5%, 1.0% and 3.0% of the bioactive ingredient at days 0, 2, 3 and 7 after the elaboration.

Fluctuations in pH and water content can be attributed to the relationship between these parameters, with changes in pH likely driven by LAB originally present in the ingredient, which affect both pH and Aw [44]. The increase in LAB, as suggested by cell counts on MRSa, is an advantageous outcome, as these vegan meat-like products, similar to their meat counterparts, often contain high levels of LAB. LAB presence in foods is generally associated with protective effects against foodborne contaminants and, in some cases, enhanced flavor profiles [45,46]. This increase in LAB concentration may be a key factor in controlling mesophilic microbial populations, typically associated with food spoilage, which might explain the minimal increase in mesophilic microorganisms in the treatment when compared to the control [47]. Despite differences in HSI values, the minimal variation suggests that treated samples and the control retained the characteristic pink hue of meat-based mortadella, as shown in Figure 2 [30]. In addition, these changes can be addressed with minimal adjustments to the recipe using low concentrations of food colorant [48]. However, since the focus of this work was to improve the microbiological stability of the mortadella using the ingredient, these modifications were not implemented. Overall, the quality of this treatment can be considered comparable to that of regular mortadella, the main goal of meat-like vegan products [49].

### 3.3. Evaluation of the Antimicrobial Properties of the Bioactive Ingredient in the Vegan Mortadellas

**Figure 3:**
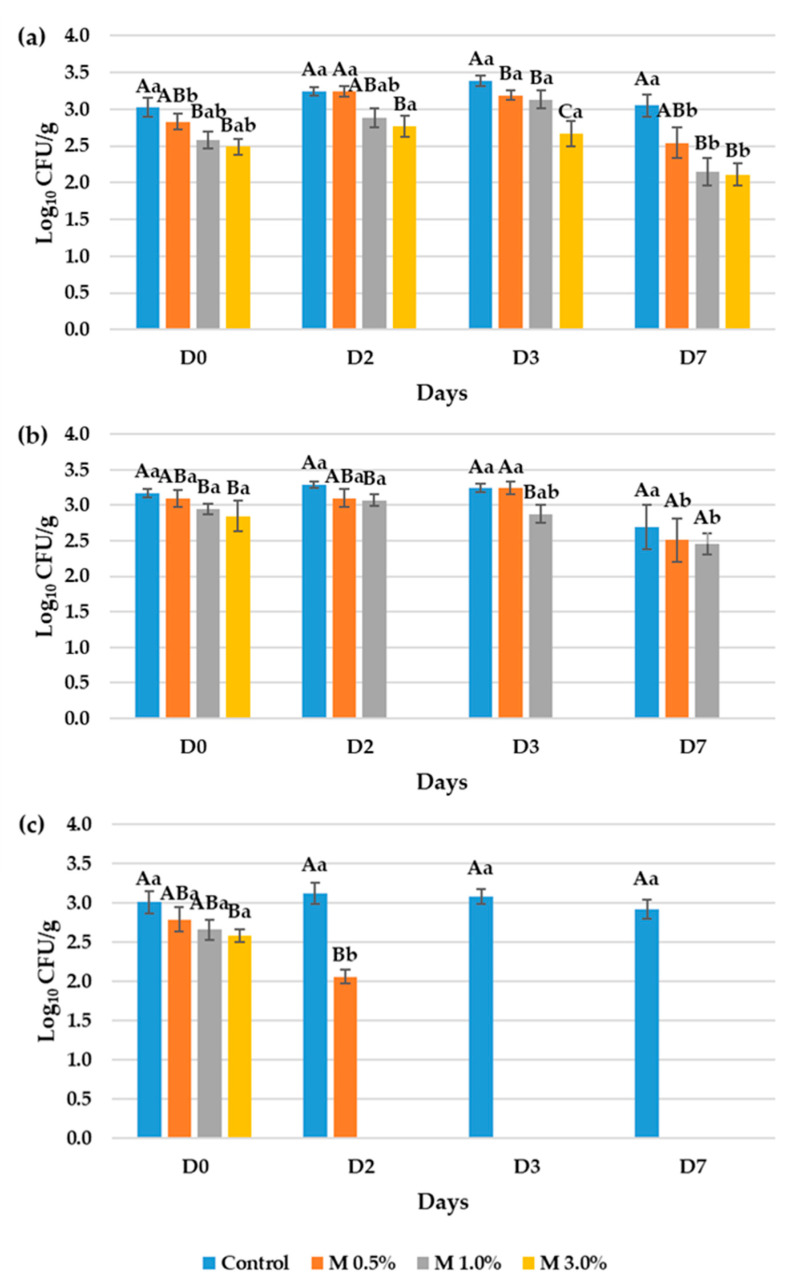
Results from the in vitro assays to evaluate the capacity of the bioactive ingredient to reduce the microbial contamination of mortadella against (**a**) *Aspergillus niger*, (**b**) *Penicillium commune* and (**c**) *Listeria monocytogenes*. The control consisted of mortadella without the bioactive ingredient, while M 0.5%, M 1.0%, and M 3.0% corresponded to mortadella samples formulated with 0.5%, 1.0%, and 3.0% of the bioactive ingredient derived from the ODN medium fermented by *L. plantarum* VC7, respectively. Different letters were used to signal significative differences of the studied parameters. Capital letters were used for the comparation among different days and case letters for the comparation among samples the same day. Results under the limit of detection (2.00 Log_10_ CFU/g) were marked as 0.00 Log_10_ CFU/g.

Figure 3 presents the results of the antimicrobial ingredient effectiveness in reducing the occurrence and proliferation of foodborne contaminants. At day 0, a significant decrease in Log_10_ CFU/g was observed across all pathogens with increasing concentrations of the bioactive ingredient. This effect was particularly notable against *Aspergillus niger* and *Listeria monocytogenes*, where the use of a 0.5% concentration of the bioactive ingredient demonstrated significant efficacy in comparison to the control. The observed reduction at day 0 in the M 0.5% samples when compared to the control was 0.19 and 0.22 Log_10_ CFU/g in the *Aspergillus niger* and *Listeria monocytogenes* assays, respectively. However, in samples contaminated with *Penicillium commune*, the significant reduction in Log_10_ CFU/g was only observed in mortadella containing 3.0% of the ingredient, where this initial decrease was of 0.32 Log_10_ CFU/g (Figure 3).

In samples contaminated with *Aspergillus niger*, fungal counts did not exceed 3.0 Log_10_ CFU/g in mortadella containing 3.0%, whereas in the rest of the samples and control fungal counts remained steady at over 3.0 Log_10_ CFU/g. *Aspergillus niger* presence at the day sevenwas 3.05, 2.54, 2.15 and 2.11 in the control sample, M 0.5%, M 1.0% and M 3.0%, respectively. The statistic alanalysis determined that samples M 1.0% and M 3.0% had significant reduction in the population of the microorganism population at the end of the assays (Figure 3a).

Assays against *Penicillium commune* showed that mortadella with higher concentrations of the ingredient reduced fungal populations below detection limits after two days of incubation, and this reduction persisted forseven days. Moreover, samples with 1.0% of the ingredient showed significant reductions in fungal populations compared to the control over a three-dayperiod of 0.36 Log_10_ CFU/g (Figure 3b).

For samples contaminated with *Listeria monocytogenes*, the treatment led to a significant decrease in microbial counts. After seven days of incubation, the pathogen was found only in the control samples. In samples treated with 0.5% of the ingredient, *L. monocytogenes* levels dropped below detection limits by day 3, and in samples with 1.0% and 3.0% of the ingredient, by day 2, while in the control samples the population of the bacterial pathogen remained on an 3.03 Log_10_ CFU/g (Figure 3c).

This data demonstrates the effectiveness of this bioactive ingredient in the biopreservation of mortadella against three common contaminants in vegan foods. The low treatment concentrations used in these assays (0.5% to 3.0%) are comparable to the typically employed in the industry for similar purposes. Companies typically use spices to prevent the contamination of vegan foods. Spices such as turmeric, mustard, ground paprika, fenugreek, basil, rosemary, and oregano, commonly used at concentrations of 0.1% to 2% [50,51]. Also, synthetic additives are the second choice for the preservation of vegan meat-like products. Nevertheless, synthetic additives are only used on limited occasions due to the strong relationship between consumer demand for vegan foods and the reduction of synthetic compounds in foods [52]. When synthetic additives are used in vegan foods, they are generally added in concentrations recommended by regulatory authorities, such as those outlined by the European Food Safety Authority. For instance, sorbic acid is permitted up to 0.2% in vegan meat- or cheese-like products, while propionic acid and its salts may be applied to cheese-like products or incorporated up to 2% [53]. Meanwhile, research increasingly focuses on novel applications of essential oils as natural antimicrobials. Extensive literature details the antimicrobial mechanisms of essential oils against foodborne pathogens and spoilage organisms. Schelz et al. (2006) provides evidence of the ability of essential oils from several plant sources to disrupt with the proper functions of gram-positive microorganism such as *L. monocytogenes* [54]. Several studies have developed direct applications of essential oils, including their use as ingredients, coatings, or external methods such as in packaging or bags that release volatile oil compounds into various food matrices [55,56,57].

The addition of spices and essential oils is more widely accepted by consumers compared to synthetic compounds of origin [58] However, these natural additives significantly impact the product taste. While consumers sometimes appreciate the flavor changes, this effect also limits their use. For example, the spiciness or garlic flavor imparted by ingredients like garlic or pepper is often unsuitable for bakery products [59,60]. In contrast, synthetic additives typically do not alter the food flavor profile when used at regulated concentrations [61,62]. Nevertheless, health concerns associated with synthetic additives have increased consumer demand for reducing their presence in foods [63] Therefore, ongoing research is needed into food additives from safe sources that have minimal impact on the flavor profile.

The bioactive ingredients developed in this study meet these requirements. LAB and oat drink are considered natural by both regulators and consumers, while the literature indicates they generate a volatile profile with fewer impactful aroma compounds compared to spices and essential oils [64,65,66]. Furthermore, there has been limited research on the development of bioactive ingredients specifically for vegan foods. Therefore, the ingredient developed here represents a novel application for the biopreservation of vegan mortadella.

## 4. Conclusions

This study demonstrated that an antifungal ingredient developed through lactic acid bacterial fermentation of oat drink has potential as an effective bioactive ingredient for vegan foods. In vitro assays showed that adding simple nutrients to the oat drink enhanced the fermentation performance, increasing the bacterial growth and antimicrobial metabolite occurrence. Additionally its antimicrobial activity, resulted in inhibition zones of 2 to over 5 mm against tested food-contaminant microorganisms, leading to its selection as a bioactive ingredient. Quality analysis of vegan mortadella containing this ingredient revealed significant differences in pH and water activity compared to the control. Microbial profiling indicated that the ingredient increased lactic acid bacterial counts while reducing natural mesophilic bacterial contamination. In shelf-life extension assays, mortadella samples with 3% of the ingredient showed significant reductions in all three contaminant microorganisms at initial measurements, suggesting that the ingredient effectively inhibits contaminant viability. In samples contaminated with *Aspergillus flavus*, fungal levels remained stable at significantly lower levels than those in the control. Additionally, assays with *Penicillium commune* and *Listeria monocytogenes* demonstrated that mortadella with 3% of the antimicrobial ingredient reduced contaminant populations below detectable limits within two days of incubation, while in control samples, contaminant levels exceeded 2.5 Log_10_ CFU/g. These findings highlight the potential of this novel ingredient for biopreserving vegan mortadella and suggest its applicability across other food matrices with similar preservation benefits.

## Figures and Tables

**Table 1:** Recipe of the different vegan mortadella developed in this study. Data was expressed in grams.

Ingredients	Samples			
	Control	M 0.5%	M 1.0%	M 3.0%
Bean mash	538.0	534.5	531.0	517.0
Water	135.0	135.0	135.0	135.0
Olive	50.0	50.0	50.0	50.0
Agar	24.4	24.4	24.4	24.4
Salt	2.0	2.0	2.0	2.0
Thyme	0.4	0.4	0.4	0.4
Garlic	0.2	0.2	0.2	0.2
Bioactive ingredient	-	3.5	7.0	21.0

**Table 2:** Results from the analysis of the fermentation parameters of the oat mediums after the 48 h incubation. The pH, bacterial count (Log_10_ LAB CFU/mL of sample), lactic acid (g/L), phenyllactic acid (mg/L).

Samples		pH	Bacterial Count	Lactic Acid	Phenyllactic Acid
Control	OD	7.39 ± 0.01 ^a^	nd	nd	nd
	ODG	6.58 ± 0.01 ^b^	nd	nd	nd
	ODN	6.41 ± 0.01 ^c^	nd	nd	nd
*L. plantarum* VC7	OD	3.44 ± 0.01 ^d^	7.37 ± 0.20 ^b^	14.84 ± 0.01 ^b^	2.61 ± 0.09 ^c^
	ODG	3.41 ± 0.02 ^e^	8.20 ± 0.09 ^a^	17.27 ± 0.01 ^a^	5.42 ± 0.21 ^b^
	ODN	3.52 ± 0.02 ^e^	8.32 ± 0.12 ^a^	17.34 ± 0.01 ^a^	9.23 ± 0.13 ^a^

Data was displayed as mean ± SD. Different letters were used to signal significative differences among each studied parameter. Results below the limit of detection were marked as nd.

**Table 3:** Results from the qualitative antimicrobial evaluation of the oat drink fermented by the *L. plantarum* VC7 isolate against different vegan mortadella contaminants.

Contaminant Microorganism	Control			*L. plantarum* VC7		
	OD	ODG	ODN	OD	ODG	ODN
*A. flavus*	-	-	-	-	++	+++
*A. fumigatus*	-	-	-	-	++	++++
*A. niger*	-	-	-	-	-	++
*A. steynii*	-	-	-	-	++	+++
*P. camemberti*	-	-	-	-	-	+++
*P. commune*	-	-	-	+	++	+++
*P. digitatum*	-	-	-	+	++	+++
*P. expansum*	-	-	-	+	+++	++++
*P. italicum*	-	-	-	-	+++	++++
*P. nordicum*	-	-	-	-	+++	++++
*P. roqueforti*	-	-	-	+	+	++
*P. verrucosum*	-	-	-	+	+++	+++
*L. monocytogenes*	-	-	-	++	++	++++

Results were expressed as “++++” when the inhibition halo was superior to 5 mm, “+++”, when the halos were among 2 to 5 mm, “++” from 1 to 2 mm, “+” lower than 1 mm and “-“ when no inhibition was observed.

**Table 4:** Results from the measurement of the (a) pH, (b) Aw, (c) moisture (g/100 g) and (d) HSI of the different mortadella samples thought the 7-day incubation.

**(a) pH**				
**Samples**	**Days**			
	**D0**	**D2**	**D3**	**D7**
Control	5.98 ± 0.03 ^BCa^	6.22 ± 0.06 ^Aa^	6.03 ± 0.01 ^Ba^	5.93 ± 0.04 ^Ca^
M 0.5%	5.70 ± 0.02 ^ABb^	5.90 ± 0.09 ^Ab^	5.68 ± 0.04 ^ABb^	5.65 ± 0.12 ^Bb^
M 1.0%	5.10 ± 0.06 ^Cc^	5.50 ± 0.13 ^Ac^	5.41 ± 0.05 ^Bc^	5.40 ± 0.03 ^Bc^
M 3.0%	4.60 ± 0.03 ^Bd^	4.85 ± 0.09 ^Ad^	4.73 ± 0.02 ^ABd^	4.74 ± 0.03 ^ABd^
**(b) Aw**				
**Samples**	**Days**			
	**D0**	**D2**	**D3**	**D7**
Control	0.975 ± 0.001 ^Ba^	0.905 ± 0.001 ^Da^	0.985 ± 0.001 ^Aa^	0.954 ± 0.001 ^Ca^
M 0.5%	0.975 ± 0.002 ^Aa^	0.907 ± 0.001 ^Da^	0.968 ± 0.002 ^Bb^	0.935 ± 0.001 ^Cb^
M 1.0%	0.925 ± 0.001 ^Bb^	0.858 ± 0.001 ^Db^	0.948 ± 0.001 ^Ad^	0.918 ± 0.001 ^Cc^
M 3.0%	0.874 ± 0.001 ^Cc^	0.862 ± 0.001 ^Db^	0.958 ± 0.001 ^Ac^	0.908 ± 0.001 ^Bd^
**(c) Moisture**				
**Samples**	**Days**			
	**D0**	**D2**	**D3**	**D7**
Control	25.22 ± 0.76 ^Bb^	25.61 ± 0.66 ^Bc^	26.34 ± 0.40 ^ABb^	28.53 ± 0.52 ^Aa^
M 0.5%	27.34 ± 0.38 ^Ab^	26.34 ± 0.31 ^Aab^	23.76 ± 1.25 ^Bb^	24.69 ± 0.97 ^Bc^
M 1.0%	27.31 ± 0.76 ^Ab^	27.74 ± 0.66 ^Aba^	24.46 ± 0.30 ^Bb^	25.01 ± 0.31 ^Ab^
M 3.0%	30.57 ± 0.19 ^Aa^	29.55 ± 0.18 ^Aa^	28.76 ± 0.96 ^Ba^	26.46 ± 0.25 ^Cb^
**(d) HSI**				
**Samples**	**Days**			
	**D0**	**D2**	**D3**	**D7**
Control	25.39 ± 4.67 ^ABc^	26.33 ± 3.85 ^Ab^	20.21 ± 0.53 ^Bc^	19.23 ± 6.54 ^ABb^
M 0.5%	49.86 ± 0.93 ^Ab^	53.09 ± 5.10 ^Aa^	43.36 ± 6.68 ^Aa^	44.17 ± 3.67 ^Aa^
M 1.0%	58.27 ± 2.91 ^Aa^	59.89 ± 4.95 ^Aa^	48.27 ± 0.46 ^Ba^	47.31 ± 5.32 ^Ba^
M 3.0%	48.13 ± 1.97 ^Ab^	52.79 ± 5.68 ^Aa^	47.01 ± 1.50 ^Aa^	44.38 ± 3.70 ^Aa^

Data was displayed as mean ± SD. Different letters were used to signal significative differences of the studied parameters. Capital letters for the comparation among different days (rows) and case letter for the comparation among samples the same day (columns).

## Data Availability

The original contributions presented in this study are included in the article. Further inquiries can be directed to the corresponding author.

## Abbreviations

The following abbreviations are used in this manuscript:

Aw	Water activity
BHI	Brain heart infusion
CFU/g	Colony forming units per gram of food
HSI	Hue, saturation, and intensity
LAB	Lactic acid bacteria
M 0.5%	Vegan mortadella with a 0.5% of the bioactive ingredient
M 1.0%	Vegan mortadella with a 1.0% of the bioactive ingredient
M 3.0%	Vegan mortadella with a 3.0% of the bioactive ingredient
MRSa	Man, Rogosa and Sharpe agar
MRSb	Man, Rogosa and Sharpe broth
PCA	Plate count agar
PDA	Potato dextrose agar
OD	Culture medium oat drink
ODG	Culture medium oat drink enriched with glucose
ODN	Culture medium oat drink enriched with nutrients
